# From Golden Rice to Golden Diets: How to turn its recent approval into practice

**DOI:** 10.1016/j.gfs.2021.100596

**Published:** 2022-03

**Authors:** Hans De Steur, Alexander J. Stein, Matty Demont

**Affiliations:** a Ghent University, Department of Agricultural Economics, Coupure Links 653, 9000, Ghent, Belgium; b European Commission, Directorate-General for Agriculture and Rural Development, 1049 Brussels, Belgium; c International Rice Research Institute, Los Baños, Laguna, Philippines

**Keywords:** Biofortification, Consumer acceptance, Golden rice, Planetary health diets, The Philippines, Vitamin A deficiency

## Abstract

Following its approval in the Philippines in July 2021, provitamin A-rich “Golden Rice” is set to become the worlds' first commercialized genetically modified crop with direct consumer benefits. Despite supplementation and fortification programs, the burden of micronutrient deficiencies remains high. For Golden Rice to be successful in reducing vitamin A deficiency, it needs to be taken up by food systems and integrated into consumer diets. Despite negative information often being associated with genetic engineering, evidence suggests that consumers react positively to Golden Rice. Thus, it offers policy makers and public health stakeholders a new, powerful option to address micronutrient malnutrition that they can integrate as a cost-effective component in broader nutrition strategies and tailor it to consumers’ heterogeneous socio-economic contexts and needs to promote “Golden Diets”. For this to happen, the right framing of the pathway from policy to consumption is crucial.

## Highlights

• Philippines' Golden Rice approval presses for moving the framing and debate forward.
• Golden Rice offers another, powerful tool to help achieve planetary health diets.
• Consumers in target countries and elsewhere overwhelmingly have positive reactions.
• Emphasizing nutritional benefits will have more impact than entering the GMO debate.
• Golden Rice needs to be integrated into food environments and nutrition strategies.

## 1 A history of controversy

July 2021 was yet another landmark for the strengthening of the agriculture-nutrition-health nexus, when the Philippines gave their green light for the cultivation of genetically modified (GM) Golden Rice, a rice variety biofortified with provitamin A (Science, 2021). Biofortification refers to the use of crop breeding or of agronomic practices to increase the mineral or vitamin content in crops to address micronutrient malnutrition and improve public health. Hundreds of conventionally biofortified crop varieties have already been released and shown impact and acceptance (Birol et al., 2015; CAST, 2020), but Golden Rice is set to become the worlds’ first commercialized GM biofortified crop.

After being declared safe for consumption in four countries (Australia, New Zealand, Canada and the United States) (Greedy, 2018), it is the Philippines that was the first to approve its cultivation, which is expected to happen in Bangladesh soon, too. Since Potrykus and Beyer developed its first version in 1999 (Qamar et al., 2020; Ye et al., 2000), Golden Rice has been facing sustained criticism that undoubtedly delayed the progress of this humanitarian project to help alleviate the health and economic burden of vitamin A deficiency (Wesseler and Zilberman, 2014). Carrying the legacy of the controversy over the widely adopted first-generation GM crops with farmer-oriented agronomic benefits (such as insect resistance or herbicide tolerance) may have intensified the struggle for approval of this second-generation GM crop with clear consumer benefits.

However, instead of looking (again) at the heated debate and polarization between proponents and opponents (Kettenburg et al., 2018), it is worthwhile to focus on how Golden Rice could be taken up by local communities and how its use could be better framed by policy makers, nutritionists and the scientific community.

## 2 The post-approval dialogue

When it comes to novel and controversial products, a routine dialogue takes place when positive new scientific evidence or policy decisions are published: pro-parties typically lend their support through positive messaging, quickly followed by anti-campaigning by opposing parties. In light of the upcoming commercialization of Golden Rice in the Philippines, the potential influence of information campaigns should not be ignored, not the least at the level of consumers (and of farmers as consumers), who are the key beneficiaries of Golden Rice.

**Fig. 1:**
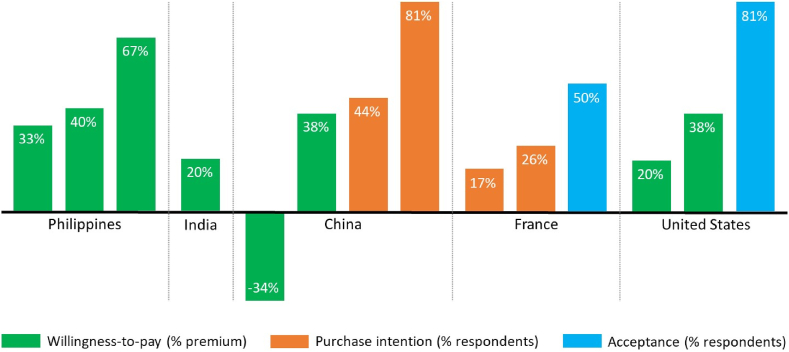
Overview of consumer acceptance studies on Golden Rice. Source: Own development, based on a systematic review, updated with recent literature (De Steur et al., 2015; Zheng et al., 2018). Note: Acceptance represents the share of consumers with a positive attitude towards Golden Rice. Purchase intention is measured through the share of consumers with an intention to purchase Golden Rice if it were available. Willingness-to-pay represents consumers' perceived added-value of Golden Rice relative to conventional rice. It is measured as the mean price premium consumers would be prepared to pay for Golden Rice—if it were available—as compared to conventional rice. Ultimately, prices are determined on the market by aggregate supply and demand and, if applicable, by government subsidies, though. (For interpretation of the references to color in this figure legend, the reader is referred to the Web version of this article.)

Information may be retained, whether it is validated or not. Translating consumer studies on Golden Rice and other GM biofortified crops into practice (De Steur et al., 2015; Zheng et al., 2018), the interplay between positive and negative information on Golden Rice can be expected to affect consumer acceptance in some way or the other. Negative information associated with genetic engineering is certainly found to reduce peoples’ intentions to consume Golden Rice, but research has shown that it would not necessarily increase overall rejection rates as long as the nutritional benefits are highlighted (De Steur et al., 2017a). This is an important finding, as it suggests that, overall, negative information does not necessarily overshadow the effect of positive information. Currently the evidence overwhelmingly points to positive consumer reactions to Golden Rice (Fig. 1), which do not differ from those reported for other GM and non-GM biofortified crops (De Steur et al., 2017b; Oparinde and Birol, 2019). Making target populations aware of the nutritional content and specific benefits of Golden Rice for their own health and well-being and that of their children could have a much larger impact than trying to resolve the larger discussion about the general benefits of genetic engineering (De Steur et al., 2017a).

This discussion often extends to the role of knowledge and education. While nutritional knowledge indeed appears to increase consumer acceptance of GM biofortified foods, the role of knowledge of genetic engineering is much less straightforward. Hence, following its approval, a new wave of communication efforts to explain the value of Golden Rice to target populations should be focused even more on its nutritional value. Thereby, the design of the communication strategy should be carefully adapted to the context, taking into account differential impacts based on the information content (e.g., length, frequency), source (e.g., trustworthiness) and channel (e.g., audiovisual, community-driven) (Birol et al., 2015).

## 3 The push for adoption

For Golden Rice to be successful in reducing vitamin A deficiency, it needs to be accepted by consumers. As studies show that changes to a biofortified crop's sensory qualities, including its color, do not act as obstacles to its acceptance (Talsma et al., 2017), communicating its nutritional value remains key. However, even if consumer acceptance is needed, and even if it can signal the market demand for Golden Rice to farmers, also adoption of the rice by the latter is important. Regardless of its potential to reduce the prevalence of vitamin A deficiency in the Philippines (e.g., nearly 1 out of 5 children of 6–59 months old in rural areas) (DOST-FNRI, 2020) and beyond (De Moura et al., 2016), agronomic performance of targeted local varieties should be at least unaffected and preferably improved. Encouragingly, earlier confined field tests of recent Golden Rice introgression lines in the Philippines and Bangladesh demonstrated both agronomic performance and carotenoid expression in three popular rice varieties (Swamy et al., 2021). The humanitarian sublicense for resource-poor farmers (i.e. farmers own the seeds, and use the seeds royalty-free without additional costs for the trait; www.goldenrice.org) could further facilitate its cultivation, as competitive seed prices will likely incentivize them to grow Golden Rice varieties (Potrykus, 2010). Indeed, the available evidence suggests that farmers will likely need to be incentivized to adopt Golden Rice (Glover et al., 2020; Stone and Glover, 2017), e.g., through higher agronomic performance and market access.

## 4 Deploying Golden Rice and moving the debate forward

When commercialized, Golden Rice needs to take its place in the current food system, i.e. it needs to be taken up by value chains and integrated into food environments (where food acquisition and consumption takes place) and consumer diets. It is important to emphasize that this crop is not, or should not, be framed as a silver bullet—or a golden bullet, as it were—for ending micronutrient malnutrition. Rather, it has an important role in addressing a particular micronutrient deficiency that still imposes a considerable burden on public health and that requires innovative but cost-effective and culturally appropriate interventions that go beyond conventional vitamin A supplementation programs that typically target high priority population groups (children of 6–59 months old) (Hamer and Keusch, 2015). Implementing food-based solutions is particularly important as these are less vulnerable to disruptions caused by funding shortfalls or catastrophes, as shown most recently by the drop in vitamin A supplementation rates due to COVID-19 (HKI, 2020). Improving the micronutrient status of poor populations can also more generally contribute to stronger immune systems, which again is of particular importance to boost their resilience in case of (public health) crises (Heck et al., 2020).

Even though disseminating Golden Rice is expected to be a valuable and cost-effective complementary intervention to combat vitamin A deficiency in particular, to win the fight against malnutrition in general, it is time to move the framing forward and shift the debate. Instead of discussing technological aspects of individual crops, such as Golden Rice, we need to come to an understanding of how to achieve “Golden Diets”, i.e. winning diets that are sustainable and wholesome.

One of the trail blazers in this respect is the EAT-Lancet Commission. It promotes a dietary shift towards “planetary health diets” that aim at striking a balance between human nutrition and planetary health (Willett et al., 2019). It acknowledges that despite nutrition programs, the burden of micronutrient deficiencies remains high and that for achieving planetary health diets, the adequacy of most micronutrients in low-income countries must increase—not least through greater consumption of fruits and vegetables, which are an essential source also of provitamin A.

However, the challenges for poor consumers are that (i) not only are their low incomes a barrier to more diversified and wholesome diets, also the relative prices of fruits and vegetables (higher compared to staple crops, such as rice) affect food consumption patterns (low intake of fruits and vegetables) and related health outcomes; and (ii) high prices and low incomes mean that wholesome diets (such as the EAT-Lancet diet) are well outside the reach of the average consumer in poor countries for the foreseeable future as they surpass their disposable daily incomes. Indeed, globally, about 3 billion people cannot afford the minimum cost of healthy diets recommended by national governments (Herforth et al., 2020). In addition to economic growth and nutrition-sensitive social protection, supply-side interventions that improve the affordability of nutritious foods are needed. This means that until widespread consumption of more diversified and wholesome diets is achieved through economic growth and nutrition-sensitive social protection, interventions that improve the affordability of nutritious foods will be crucial (Fan et al., 2021).

In this context, Golden Rice has a valuable role to play. It can be a provitamin A-rich component in broader interventions that rely on rice being a traditional and accepted staple to enable more nutrient-rich diets that better satisfy consumers’ physiological and nutritional needs than diets that are based on conventional rice. Such diets can still fit the respective socio-demographic, economic and cultural contexts and fulfill the hedonic motivations of the target groups (Custodio et al., 2021).

**Fig. 2:**
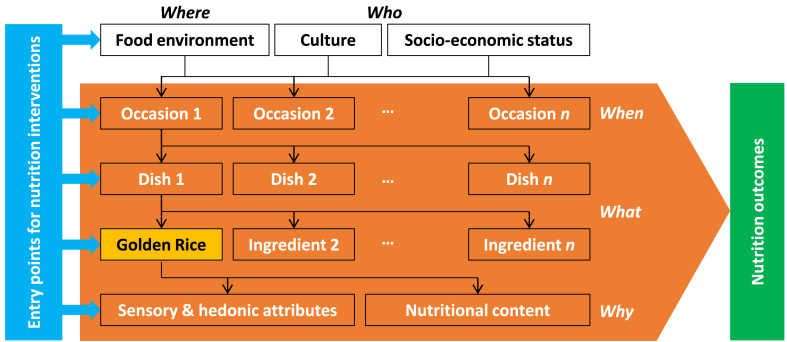
Integrating Golden Rice as a component of a “Golden Diet.” Source: adapted from Custodio et al. (2021). Note: Diets are structured through culturally inherited “gastronomic systems” that are contextualized by the socio-economic status of consumers (*who*) and the food environment (*where*) in which they purchase and consume food. The gastronomic system defines the eating occasions (*when*) during which food is typically consumed (breakfast, snacks, lunch, dinner, special occasions, etc.), which in their turn determine the dishes (*what*) that are consumed (rice-based and other dishes), which combine ingredients (rice, staples, vegetables, viands, sauces, etc.) that carry certain sensory and hedonic attributes and provide nutritional content (*why*). Each of the hierarchical layers in the system provides entry points for nutrition interventions. For example, Golden Rice can be promoted in food environments (*where*; e.g., in schools, cafeteria, etc.), during certain occasions (*when*; e.g., as a healthy breakfast, snack, lunch or dinner), through special dishes centered around Golden Rice or as a healthy ingredient in rice-based dishes (*what*), and for its sensory, hedonic and nutritional attributes (*why*; e.g., through information campaigns, school education programs, etc.). (For interpretation of the references to color in this figure legend, the reader is referred to the Web version of this article.)

Once available on the market, Golden Rice will have to find its place within consumer diets, it will have to be paired to other ingredients in terms of its various sensory attributes (aroma, taste, color), and it will need to be incorporated into existing dishes or become the center piece of new ones. These dishes, in turn, will have to become integrated in eating occasions (breakfast, snacks, lunch, dinner, and special occasions), which are determined by consumers’ culture, their socio-economic status and the food environments they are exposed to. All these components—the *where, who, when, what, and why*—are part of a system that needs to be optimized to achieve “Golden Diets” (Fig. 2).

The system provides multiple entry points for nutrition interventions to help consumers achieve such diets. For these interventions to have impact, they should be situation-specific and use multiple strategies that need to be tailored to consumers’ heterogeneous situations and needs (Custodio et al., 2021); e.g., using Golden Rice to diversify micronutrient strategies, or as a provitamin A-rich food item when promoting nutrient-richer diets to resource-poor target groups.

After a long series of delays in the regulatory process, the approval of Golden Rice in the Philippines marks an important breakthrough in the fight against vitamin A deficiency. Policy makers and public health stakeholders now have a new, powerful option to help target populations achieve planetary health diets. However, the success of Golden Rice and other nutritionally enhanced crops in the pipeline will crucially depend on two factors: (i) how these crops are integrated into national nutrition strategies; and (ii) how consumers manage to incorporate them into their daily lives in their efforts to achieve “Golden Diets.” Such diets can be another illustration on how to operationalize the EAT-Lancet planetary health diets (Béné et al., 2020), but it is up to policy makers to ensure that these crops are integrated as cost-effective components within a broader nutrition strategy, and to communicate to consumers that eating Golden Diets means winning.

## Disclaimer

The information and views set out in this article are those of the authors and do not necessarily reflect the official position of the European Commission.

## Author contributions

HDS: conceptualization, formal analysis, investigation, methodology, visualization, writing—original draft, writing—review & editing; AJS: formal analysis, investigation, methodology, visualization, writing—review & editing; MD: conceptualization, formal analysis, investigation, methodology, visualization, writing—review & editing.

## Declaration of competing interest

The authors declare that they have no known competing financial interests or personal relationships that could have appeared to influence the work reported in this paper.

## References

[bib1] Béné C., Fanzo J., Haddad L. (2020). Five priorities to operationalize the EAT–Lancet Commission report. Nat. Food.

[bib2] Birol E., Meenakshi J.V., Oparinde A., Perez S., Tomlins K. (2015). Developing country consumers’ acceptance of biofortified foods: a synthesis. Food Sec..

[bib3] CAST (2020). Food biofortification: Reaping the benefits of science to overcome hidden hunger. https://www.cast-science.org/publication/food-biofortification-reaping-the-benefits-of-science-to-overcomehidden-hunger/.

[bib4] Custodio M.C., Ynion J., Samaddar A. (2021). Unraveling heterogeneity of consumers’ food choice. Glob. Food. Sec..

[bib5] De Moura F.F., Moursi M., Donahue Angel M. (2016). Biofortified β-carotene rice improves vitamin A intake and reduces the prevalence of inadequacy among women and young children in a simulated analysis in Bangladesh, Indonesia, and the Philippines. Am. J. Clin. Nutr..

[bib6] De Steur H., Blancquaert D., Strobbe S. (2015). Status and market potential of transgenic biofortified crops. Nat. Biotechnol..

[bib7] De Steur H., Demont M., Gellynck X., Stein A.J. (2017). The social and economic impact of biofortification through genetic modification. Curr. Opin. Biotechnol..

[bib8] De Steur H., Wesana J., Blancquaert D., Van Der Straeten D., Gellynck X. (2017). Methods matter: a meta-regression on the determinants of willingness-to-pay studies on biofortified foods. Ann. N. Y. Acad. Sci. ANYAS.

[bib9] DOST-FNRI (2020).

[bib10] Fan S., Headey D., Rue C., Thomas T. (2021). Food systems for human and planetary health: economic perspectives. Annu. Rev. Resour. Econ..

[bib11] Glover D., Kim S.K., Stone G.D. (2020). Golden Rice and technology adoption theory: a study of seed choice dynamics among rice growers in the Philippines. Technol. Soc..

[bib12] Greedy D. (2018). Golden Rice is safe to eat, says FDA. Nat. Biotechnol..

[bib13] Hamer D.H., Keusch G.T. (2015). Vitamin A deficiency: slow progress towards elimination. Lancet Glob. Health.

[bib14] Heck S., Campos H., Barker I. (2020). Resilient agri-food systems for nutrition amidst COVID-19: evidence and lessons from food-based approaches to overcome micronutrient deficiency and rebuild livelihoods after crises. Food Sec..

[bib15] Herforth A., Bai Y., Venkat A., Mahrt K., Ebel A., Masters W.A. (2020).

[bib16] HKI (2020). Building children’s immune systems in Kenya in the time of COVID-19. https://www.hki.org/our-stories/building-childrens-immune-systems-in-kenya-in-the-time-of-covid-19/.

[bib17] Kettenburg A.J., Hanspach J., Abson D.J., Fischer J. (2018). From disagreements to dialogue: unpacking the Golden Rice debate. Sust. Sci..

[bib18] Oparinde A., Birol E., Ferranti P. (2019). Encyclopedia of Food Security and Sustainability.

[bib19] Potrykus I. (2010). Lessons from the ‘humanitarian golden rice’ project. N. Biotechnol..

[bib20] Qamar S., Tantray A.Y., Bashir S.S., Zaid A., Wani S.H. (2020). Genomics and Genetic Engineering.

[bib21] Science (2021). Golden rice to sprout in the Philippines. Science.

[bib22] Stone G.D., Glover D. (2017). Disembedding grain: golden rice, the green revolution, and heirloom seeds in the Philippines. Agric. Hum. Val..

[bib23] Swamy B.M., Marundan S., Samia M., Ordonio R.L. (2021). Development and characterization of GR2E Golden rice introgression lines. Sci. Rep..

[bib24] Talsma E.F., Melse-Boonstra A., Brouwer I.D. (2017). Acceptance and adoption of biofortified crops in low- and middle-income countries: a systematic review. Nutr. Rev..

[bib25] Wesseler J., Zilberman D. (2014). The economic power of the Golden Rice opposition. Environ. Dev. Econ..

[bib26] Willett W., Rockström J., Loken B. (2019). Food in the Anthropocene: the EAT–Lancet Commission on healthy diets from sustainable food systems. Lancet.

[bib27] Ye X., Al-Babili S., Klöti A., Zhang J., Lucca P., Beyer P., Potrykus I. (2000). Engineering the provitamin A (β-carotene) biosynthetic pathway into (carotenoid-free) rice endosperm. Science.

[bib28] Zheng Z., Henneberry S.R., Sun C., Nayga R.M. (2018). Consumer demand for genetically modified rice in urban China. J. Agric. Econ..

